# Upfront Cytoreductive Surgery as a Bridge to Systemic Therapy for Recurrent Peritoneal Dissemination of Ovarian Angiosarcoma: A Pediatric Case Report

**DOI:** 10.70352/scrj.cr.26-0128

**Published:** 2026-05-16

**Authors:** Masanaga Matsumoto, Takato Sasaki, Yudai Goto, Toko Shinkai, Hiroko Fukushima, Hidetoshi Takada, Ayumi Shikama, Yusuke Kobayashi, Daisuke Miyazawa, Mari Watanabe, Daisuke Matsubara, Kouji Masumoto

**Affiliations:** 1 Department of Pediatric Surgery, Institute of Medicine, University of Tsukuba, Tsukuba, Ibaraki, Japan; 2 Department of Child Health, Institute of Medicine, University of Tsukuba, Tsukuba, Ibaraki, Japan; 3 Department of Obstetrics and Gynecology, Institute of Medicine, University of Tsukuba, Tsukuba, Ibaraki, Japan; 4 Department of Diagnostic Pathology, Institute of Medicine, University of Tsukuba, Tsukuba, Ibaraki, Japan

**Keywords:** ovarian angiosarcoma, cytoreductive surgery, recurrent, peritoneal dissemination, malignant ascites, children

## Abstract

**INTRODUCTION:**

Ovarian angiosarcoma is exceptionally rare, especially considering those few pediatric cases that have been reported, and displays a poor outcome. Recurrent peritoneal dissemination of ovarian angiosarcoma is generally managed with systemic therapy, but refractory malignant ascites may compromise performance status and hinder treatment delivery. Herein, we report a pediatric case in which upfront cytoreductive surgery (CRS) controlled refractory ascites and enabled subsequent systemic therapy for recurrent peritoneal dissemination.

**CASE PRESENTATION:**

A 14-year-old girl presented with acute abdominal pain and underwent left salpingo-oophorectomy for a large ovarian tumor. Histopathological examination revealed mucinous carcinoma occupying most of the mass, with focal areas containing angiosarcoma and mature teratoma components. Staging surgery 1 month later showed no residual malignancy, and no adjuvant chemotherapy was administered. Six months after the initial surgery, she developed diffuse peritoneal dissemination and massive hemorrhagic ascites. Paracentesis provided only transient relief; ascites rapidly reaccumulated, oral intake became impossible, and progressive anemia required daily red blood cell transfusions for 6 consecutive days. Cell-block cytology of ascitic fluid suggested angiosarcoma. As adequate ascites control was not achievable and chemotherapy was considered impracticable, upfront CRS was performed, consisting of a total hysterectomy with right salpingo-oophorectomy and debulking of peritoneal dissemination. Resected lesions were exclusively composed of angiosarcoma. Postoperatively, the ascites markedly decreased, enabling the initiation of chemotherapy on POD 7 and completion of adjuvant chemoradiotherapy without substantial interruption. The patient has remained free of radiologic disease and ascites for 1 year after the initial surgery.

**CONCLUSIONS:**

This report demonstrated that CRS could provide effective control of malignant hemorrhagic ascites in recurrent peritoneal dissemination of ovarian angiosarcoma. Upfront CRS can be considered as a potential initial treatment option, facilitating the timely delivery and completion of subsequent systemic therapy.

## Abbreviations

COG
: Children’s Oncology Group
CRS
: cytoreductive surgery
EpSSG
: European pediatric Soft Tissue Sarcoma Study Group
HIPEC
: hyperthermic intraperitoneal chemotherapy
NRSTS
: non-rhabdomyosarcoma soft tissue sarcoma

## INTRODUCTION

Ovarian angiosarcoma is exceptionally rare among primary ovarian cancers, with approximately 60 cases documented in the literature,^1)^ and only 5 cases have been reported in children as far as we have reviewed.^2–6)^ Most patients with ovarian angiosarcoma initially present at advanced stages^2,7)^ and the prognosis is dismal despite multimodal therapy; the 5-year overall survival is reported as <30%, even in nonmetastatic disease, and metastatic presentation is typically associated with rapid progression and death within approximately 1 year.^8)^

Owing to its rarity, a standardized treatment strategy for ovarian angiosarcoma has not yet been established. Recent literature reviews have mainly focused on initial management with radical surgery, chemotherapy, and radiotherapy, with little guidance for the management of recurrent disease.^9,10)^ Intraperitoneal relapse of ovarian angiosarcoma or other sarcomas is generally managed with upfront systemic chemotherapy in pediatric cases.^11,12)^ However, patients with peritoneal dissemination of ovarian angiosarcoma frequently develop massive hemorrhagic ascites, leading to a poor performance status, progressive anemia,^2)^ and the discontinuation or omission of systemic chemotherapy.^13)^ In gynecologic oncology, CRS is a common surgical strategy aimed at maximal feasible tumor debulking. Although commonly performed after chemotherapy, CRS might contribute to the control of malignant ascites.^14)^

Herein, we present the case of a pediatric patient with recurrent peritoneal dissemination of an ovarian angiosarcoma with refractory hemorrhagic ascites. Upfront CRS allowed for the sufficient control of the refractory ascites and contributed as a bridge to subsequent chemoradiotherapy.

## CASE PRESENTATION

**Fig. 1:**
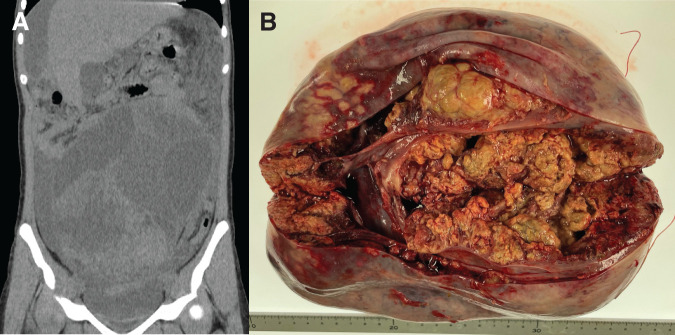
Perioperative findings at the initial surgery. (**A**) Abdominal CT shows a large multilocular cystic pelvic mass with a “stained-glass appearance” with solid components (total size: 22.4 × 17.6 × 10.9 cm; solid component size: 14.4 × 13.8 × 9.5 cm) and massive ascites, initially suggesting an adnexal torsion. (**B**) Gross cut surface of the resected left ovarian tumor (total size: 19.1 × 11.9 × 10.4 cm) showing predominantly confluent multinodular and papillary solid components associated with a thickened cyst wall, which constituted the majority of the tumor.

**Fig. 2:**
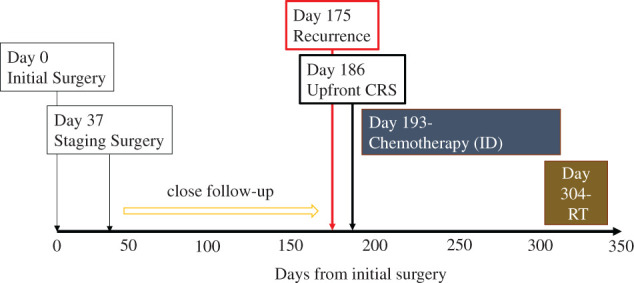
Clinical course of the present case after initial surgery. CRS, cytoreductive surgery; ID, ifosfamide and doxorubicin; RT, radiotherapy

A 14-year-old girl with no significant past medical history except for asthma was referred to our hospital due to acute abdominal pain. A large pelvic mass was identified on abdominal CT performed at the referring hospital. The patient had a 2-year history of progressive abdominal distension and a recent 7-kg weight loss over 1 month. She was 158 cm tall and weighed 54.4 kg. On physical examination, a large abdominal mass occupying most of the abdomen was palpable without signs of peritoneal irritation. Laboratory examination revealed anemia (hemoglobin, 8.9 g/dL) and an increased inflammatory marker (i.e., C-reactive protein, 4.86 mg/dL) level. Tumor marker evaluation indicated high cancer antigen-125 (CA-125) (268 U/mL [reference range, <35 U/mL]) and lactate dehydrogenase (321 U/L) expressions, whereas other serum tumor markers, including carcinoembryonic antigen, CA19-9, alpha-fetoprotein, and β-human chorionic gonadotropin, remained within the normal range. CT revealed a large multilocular abdominal cystic mass with solid components (total mass size 22.4 × 17.6 × 10.9 cm) and massive ascites, suspecting adnexal torsion (**Fig. 1A**). Emergent surgery, performed late at night, revealed that the left ovary had been completely replaced by a large cystic mass and nonhemorrhagic, straw-colored ascitic fluid was present without evidence of adnexal torsion. Based on previously reported techniques^15)^ and with careful consideration of cosmetic outcomes, a Pfannenstiel incision was selected for surgical access. The cyst contents were carefully aspirated (approximately 1000 mL) without macroscopic spillage, using a ring wound retractor, cyanoacrylate adhesive, and an adhesive incise drape, followed by a left salpingo-oophorectomy (**Fig. 1B**). The operative time was 3 h and 24 min, and the estimated intraoperative blood loss was negligible. Histopathological analysis revealed that the left ovarian tumor consisted of 3 contiguous components: mucinous carcinoma, which occupied the vast majority of the mass, with angiosarcoma and mature teratoma confined to a small focal area within the tumor. On cytological examination, the aspirated cyst and ascitic fluid obtained immediately after entering the abdominal cavity were both negative for malignancy. The postoperative course is shown in **Fig. 2**.

Follow-up abdominal ultrasonography revealed a mass in the right ovary, and subsequent MRI suggested a mature ovarian teratoma. One month later, the patient underwent right ovarian tumor enucleation along with comprehensive staging surgery for the left ovarian tumor, including appendectomy, partial omentectomy, peritoneal biopsies, and repeat cytological examination of ascitic fluid. The right ovarian tumor was diagnosed as a mature teratoma without malignant components, and the results in all additional staging biopsies were negative for malignancy. Based on these findings, the mucinous carcinoma was classified as stage IA according to the FIGO 2014 classification, and angiosarcoma was categorized as a low-risk NRSTS falling into the surgery-alone group in the COG Study ARST0332^16)^ and EpSSG NRSTS protocols^17)^ and regarded as stage IA according to the FIGO 2014 classification. Therefore, no adjuvant chemotherapy was administered.

**Fig. 3:**
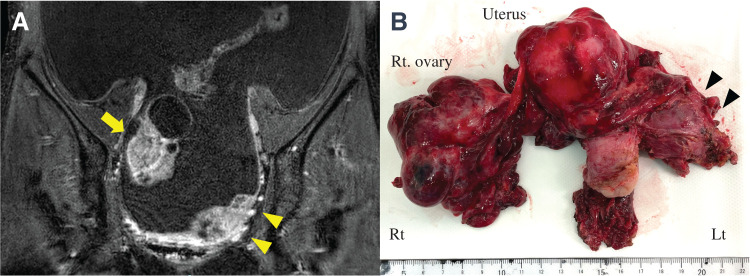
Preoperative imaging and gross surgical findings of recurrent peritoneal dissemination of ovarian angiosarcoma. (**A**) Pelvic MRI reveals diffuse peritoneal dissemination involving the left pouch of Douglas (arrowheads), uterine serosa, and right adnexa (arrow). (**B**) Macroscopic view of the resected uterus, right adnexa, and peritoneal lesions at the left pouch of Douglas (arrowheads) showing multiple hemorrhagic nodules consistent with disseminated angiosarcoma.

**Fig. 4:**
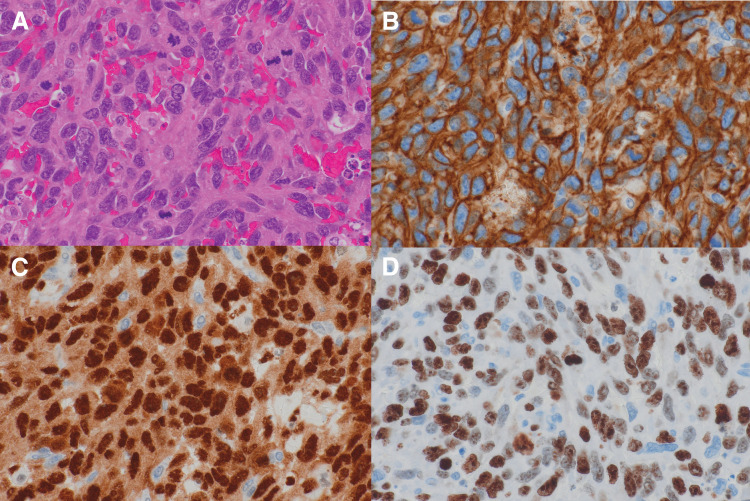
Histopathological findings of recurrent angiosarcoma dissemination. (**A**) Hematoxylin–eosin staining shows solid vascular proliferations of atypical endothelial cells without residual mucinous carcinoma or teratomatous components (×400). (**B**) Immunohistochemistry for CD31 highlights diffuse membranous staining of the neoplastic endothelial cells (×400). (**C**) ERG immunostaining shows strong nuclear positivity in the tumor cells (×400). (**D**) Ki-67 immunostaining reveals a very high proliferative index of approximately 90% (×400).

Six months after the initial surgery, she presented with extremely rapid abdominal distension. MRI revealed diffuse intra-abdominal dissemination involving the left pouch of Douglas, uterus, and right ovary with massive ascites (**Fig. 3A**). Therapeutic paracentesis with drainage of 1 L of hemorrhagic ascitic fluid provided only transient symptomatic relief. Ascitic fluid rapidly reaccumulated the following day and an additional 2 L was drained 2 days later. Cell-block cytology of the ascitic fluid suggested angiosarcoma. Ascites control remained inadequate, and severe abdominal distension due to massive ascites markedly impaired oral intake despite the absence of bowel obstruction. In addition, her anemia progressed, and she required daily red blood cell transfusions for 6 consecutive days following the second paracentesis. In consultation with gynecologists, we decided to proceed with CRS as the initial treatment for recurrence, anticipating improved control of the malignant hemorrhagic ascites, because the completion of upfront systemic chemotherapy was considered impracticable. The patient underwent a total hysterectomy with right salpingo-oophorectomy and debulking of the peritoneal dissemination (**Fig. 3B**), while the serosal implants on the bowel were intentionally left unresected to minimize the surgical invasiveness. The operative time was 5 h and 10 min, and the intraoperative blood loss, including ascitic fluid, was 1800 mL. Histopathological examination demonstrated that all metastatic lesions were composed exclusively of angiosarcoma without mucinous carcinoma or teratoma components (**Fig. 4A**). Immunohistochemical analysis showed positivity for CD31 and ERG (**Fig. 4B** and **4C**), and the tumor exhibited very high proliferative activity, with a Ki-67 labeling index of approximately 90% (**Fig. 4D**).

Postoperatively, the hemorrhagic ascites decreased markedly, no further blood transfusions were required, and her oral intake rapidly improved. Systemic chemotherapy with ifosfamide and doxorubicin, based on EpSSG NRSTS protocols,^17)^ was initiated on POD 7, and the patient subsequently completed adjuvant chemoradiotherapy without substantial interruption. At the most recent follow-up, 1 year after the initial surgery and 6 months after CRS, the patient remained free of radiologic evidence of a tumor or ascites.

## DISCUSSION

In the present pediatric case of ovarian angiosarcoma, an exceedingly rare tumor, we experienced early postoperative recurrence and subsequent multimodal treatment including upfront CRS, contributing to the control of hemorrhagic ascites caused by recurrent peritoneal dissemination and serving as a bridge to systemic therapy. Moreover, in this report, we summarize previously reported stage I ovarian angiosarcoma and discuss considerations related to the initial treatment strategy including surgical decision-making.

To date, only 10 cases of stage I ovarian angiosarcoma have been published with adequately documented follow-up,^2,7,10,18–23)^ including 1 pediatric case^2)^ (**Table 1**). Recurrence was reported in 5 of the 10 patients within 3–18 months after the initial surgery. Four relapses presented as diffuse peritoneal dissemination,^2,19–21)^ and 1 as lung metastasis.^18)^ Despite this relatively high recurrence rate, no disease-specific clinical guidelines have been established due to the rarity of this tumor. According to the COG Study ARST0332^16)^ and the EpSSG NRSTS protocols,^17)^ such tumors might be categorized as low-risk NRSTS, for which observation after complete resection without adjuvant chemotherapy could be considered. The role of postoperative adjuvant therapy for stage I ovarian angiosarcoma might warrant further consideration.

**Table 1:** Previous reported cases of stage I ovarian angiosarcoma

Author	Year	Age	Initial surgery	Adjuvant systemic therapy	Recurrence	Treatment for recurrence	Status	Survival duration from diagnosis (months)
Lifschitz-Mercer et al.^20)^	1998	25	USO	Ifosfamide, doxorubicin	+ (18 months)	NA	Alive	18
Furihata et al.^18)^	1998	46	TAH + BSO + PLND	Cisplatin, RT	+ (4 months)	Chemotherapy	DOD	9
Nucci et al.^21)^	1998	27	USO	—	—	—	NED	14
Nucci et al.^21)^	1998	42	USO	NA	NA	NA	DOD	<24
Quesenberry et al.^22)^	2005	31	TAH + BSO	MAID	—	—	NED	10
Bösmüller et al.^23)^	2011	81	TAH + USO (post-USO)	Doxorubicin	—	—	NED	11
Pariury et al.^2)^	2019	11	USO	—	+ (6 months)	Chemotherapy CRS + HIPEC	Alive	39
Ye et al.^7)^	2021	47	TAH + BSO + PLND	Olaparib, immunotherapy, RT	—	—	NED	9
Zhou et al.^19)^	2023	41	TAH + BSO	MAID	+ (3 months)	Chemotherapy Immunotherapy	Alive	27
Johnson and Argenta^10)^	2023	29	BSO (post-TAH)	Olaparib	—	—	NED	20
Matsumoto (our case)	2026	14	USO	—	+ (6 months)	CRS chemotherapy, RT	Alive	12

BSO, bilateral salpingo-oophorectomy; CRS, cytoreductive surgery; DOD, dead of disease; HIPEC, hyperthermic intraperitoneal chemotherapy; MAID, mesna, doxorubicin, ifosfamide and dacarbazine; NA, not available; NED, no evidence of disease; PLND, pelvic lymph node dissection; RT, radiotherapy; TAH, total abdominal hysterectomy; USO, unilateral salpingo-oophorectomy

In the present case, the patient was managed with a stage I disease due to the results of the oncological examination, despite intraoperative decompression of a large ovarian cystic mass with solid components. No malignant cells were detected in the aspirated cyst and ascitic fluids or all specimens obtained at the subsequent staging laparotomy 1 month later. Based on these findings and multidisciplinary discussion, adjuvant chemotherapy was not administered. Watson et al.^15)^ reported cyst aspiration as a technique to improve cosmetic outcomes. However, all cases were discussed preoperatively at a multidisciplinary tumor board, and masses suspected to be neoplastic were excluded from this approach. As our initial operation was performed emergently late at night, the operative approach was selected without sufficient preoperative multidisciplinary discussion. This case underscores the need for caution when considering intraoperative decompression of a large ovarian mass with solid components, even in an emergency setting.

In the present case, CRS served as a successful control of progressive anemia due to malignant hemorrhagic ascites caused by recurrent peritoneal dissemination of ovarian angiosarcoma. Refractory malignant ascites is typically managed with repeated therapeutic paracentesis to palliate symptoms, and chemotherapy is initiated when feasible.^14)^ However, malignant ascites secondary to peritoneal disseminated angiosarcoma is frequently hemorrhagic and is often associated with progressive anemia^2)^; therefore, repeated drainage often necessitates transfusion support and undermines treatment continuity. In the present case, ascitic fluid rapidly reaccumulated after the initial paracentesis, requiring repeat drainage 2 days later, and her anemia progressed concurrently, leading to the conclusion that maintaining systemic chemotherapy would be difficult. Reduction in peritoneal tumor bulk by CRS is known to be mostly associated with a reduction in ascites^14)^; thus, we prioritized upfront CRS before chemotherapy. Recently, CRS combined with HIPEC has been explored in selected pediatric malignancies with peritoneal dissemination.^12)^ However, its role in angiosarcoma remains unclear and typically requires favorable response to neoadjuvant chemotherapy.^2,24)^ In our patient, due to the need for immediate symptomatic control, upfront CRS alone was considered the most pragmatic strategy to enable subsequent systemic therapy.

There is still no universally accepted definition regarding “CRS”; therefore, when peritoneal dissemination is extensive, the extent of resection and balance between completeness and invasiveness inevitably depend on the surgeon’s judgment. In the present case, we removed the largest implant from the left pouch of Douglas en bloc with the surrounding peritoneum, uterus, and right adnexa, and excised all the resectable parietal peritoneal lesions as completely as possible, while deliberately avoiding resection of the serosal implants on the bowel. Despite this limited approach, we achieved clinically meaningful control of the malignant ascites, enabling the early initiation and completion of systemic chemotherapy. Because excessively aggressive CRS may increase perioperative morbidity and delay systemic treatment, a goal-directed, complication-sparing CRS strategy aimed at securing a therapeutic window for chemotherapy may represent a reasonable approach in select patients.

## CONCLUSIONS

This pediatric case suggests that upfront CRS, as an initial strategy for recurrent peritoneal dissemination of ovarian angiosarcoma, can effectively control malignant hemorrhagic ascites and facilitate the completion of planned systemic therapy.

## DECLARATIONS

### Availability of data and materials

The datasets supporting the conclusions of this article are included within the article.
